# Modulating Myofibroblastic Differentiation of Fibroblasts through Actin-MRTF Signaling Axis by Micropatterned Surfaces for Suppressed Implant-Induced Fibrosis

**DOI:** 10.34133/research.0049

**Published:** 2023-02-21

**Authors:** Weiju Han, Qi Chu, Junliang Li, Zixuan Dong, Xuetao Shi, Xiaoling Fu

**Affiliations:** ^1^ School of Materials Science and Engineering, South China University of Technology, Guangzhou 510006, P. R. China.; ^2^ National Engineering Research Center for Tissue Restoration and Reconstruction and Innovation Center for Tissue Restoration and Reconstruction, Guangzhou 510006, P. R. China.; ^3^ Laboratory of Biomedical Engineering of Guangdong Province, South China University of Technology, Guangzhou 510006, P. R. China.; ^4^ School of Biomedical Sciences and Engineering, South China University of Technology, Guangzhou International Campus, Guangzhou 511442, P. R. China.

## Abstract

Myofibroblasts, the primary effector cells for implant-induced fibrosis, contribute to this process by secreting excessive collagen-rich matrix and contracting. Thus, approaches that suppress myofibroblasts may achieve desirable suppression effects in the fibrotic process. As one of the important physical properties of materials, material topographical structures have been proven to affect various aspects of cell behaviors, so is it possible to manipulate the formation of myofibroblasts by tailoring the topographical properties of medical devices? In this study, polycaprolactone (PCL) surfaces with typical micropatterns (micro column and micro pit) were fabricated. The regulatory effects of surface micropatterns on the myofibroblastic differentiation of fibroblasts were investigated. Compared to the flat surfaces and surfaces with micro pit, surfaces with micro columns triggered the F- to G-actin transition, inhibiting the nuclear transfer of myocardin-related transcription factor-A. Subsequently, the downstream gene α-smooth muscle actin, which is a marker of myofibroblasts, was suppressed. Further in vivo investigation showed that PCL implants with micro-column-patterned surfaces inhibited the formation of peri-implant fibrotic capsules. Our results demonstrate that surface topographical properties are a potent regulator of fibroblast differentiation into myofibroblasts and highlight the antifibrotic potential of modifying surfaces with micro-column patterns.

## Introduction

With the fast-growing use of implanted medical devices, the inhibition of implant-induced fibrosis has become a major issue to resolve [1]. After the implant enters the host, it will cause a complex foreign body reaction [2], which ultimately leads to the fibrotic encapsulation of the implant, causing implant failure. Similar to organ fibrosis, myofibroblasts, a smooth-muscle-like contractile cell type that transformed from fibroblasts (FBs), are believed to be a key trigger for the implant-induced fibrotic reaction [3–6]. Therefore, approaches that suppress myofibroblasts may achieve desirable suppression effects in the fibrotic process.

Theoretically, the regulation of all physiological events involved in myofibroblast formation, activation, inactivation, and apoptosis has the potential to treat fibrosis [7–12]. Small-molecule inhibitors and antibodies to specific targets in fibrosis-related signaling are often used. However, this approach is likely to cause side effects due to the pleiotropy of these molecules [13–15]. The use of small interfering RNA gene silencing as a promising treatment is still in the early stage of development, complicated, and expensive [16,17].

With the unveiling of the complex interaction between materials and host tissues/cells, modulating myofibroblasts by tailoring the physical properties of materials has attracted increasing attention [18–20]. For example, material stiffness has been widely recognized as a vital factor that triggers the myofibroblastic differentiation of FBs [21,22]. Noskovicova et al. [23] demonstrated that softening of the implant surfaces by coating a skin-soft silicone layer suppressed myofibroblast activation and reduced the formation of fibrotic capsules around subcutaneous implants in mice. As one of the important physical properties of materials, material topological structure also greatly affects cell behaviors. Kyle et al. [24] reported that biomimetic textured silicone surfaces with acellular dermal matrix topographical cues attenuated the acute in vitro foreign body reaction of FBs to silicone. In addition to the topographical cues inspired by the native extracellular matrix, simple regular topographic micropatterns exhibited similar regulatory effects on cells. Kim et al. [25] found that microgroove structures with different densities promoted FB migration and extracellular matrix deposition. Our previous study showed that anisotropic nanofibers enhanced the myofibroblastic differentiation of skin FBs by activating integrin β1 signaling [26]. Therefore, identifying specific topographic micropatterned surfaces that inhibit FBs from differentiating into myofibroblasts and reduce the fibrotic encapsulation of implants may provide valuable clues to resolving implant-induced fibrosis.

Currently, most studies on implant-induced fibrosis choose silicone as a material model; however, with the advancement of material science and technology, an increasing number of medical devices have been fabricated with biocompatible and degradable materials. Polycaprolactone (PCL) is one of the most commonly used Food and Drug Administration-approved polymers because of its tunable biodegradability and excellent biocompatibility. Currently, it has been utilized as the raw material of novel absorbable medical devices [27–29]. Thus, in this study, PCL surfaces with typical microstructures (flat, micro column [MC], and micro pit [MP]) were fabricated by combining photolithography and casting. The size of MC was chosen to be 5 μm in diameter and 6 μm in height for 3 reasons. First and foremost, it has been reported that cultured myofibroblasts form long “supernatural” focal adhesions (FAs) (8 to 30 μm), while the FAs in α-smooth muscle actin (α-SMA)-negative FBs are smaller “classical” ones (2 to 6 μm) [30]. Thus, the 5-μm-diameter MC may inhibit the formation of myofibroblasts by restricting their FA size. Second, MC with this size does not deform elastically, which means that all surfaces in this study have the same elasticity [31]. Therefore, the potential effect of different elasticity on cell differentiation is excluded [32]. Besides, the height of the column is larger than the thickness of a FB (≤4 μm) [33]. A MP with the same size was set as a mirror structure of the MC. The effects of these micropatterns on the myofibroblastic differentiation of FBs, together with their modulation of the actin-myocardin-related transcription factor (MRTF) signaling axis, a key regulatory signaling pathway involved in this process, were investigated. Furthermore, the implant-induced fibrosis induced by implants with different micropatterned surfaces was evaluated through a rat subcutaneous implant model.

## Results and Discussion

### Micropatterned PCL surfaces induced different morphologies of FBs

**Fig. 1.**
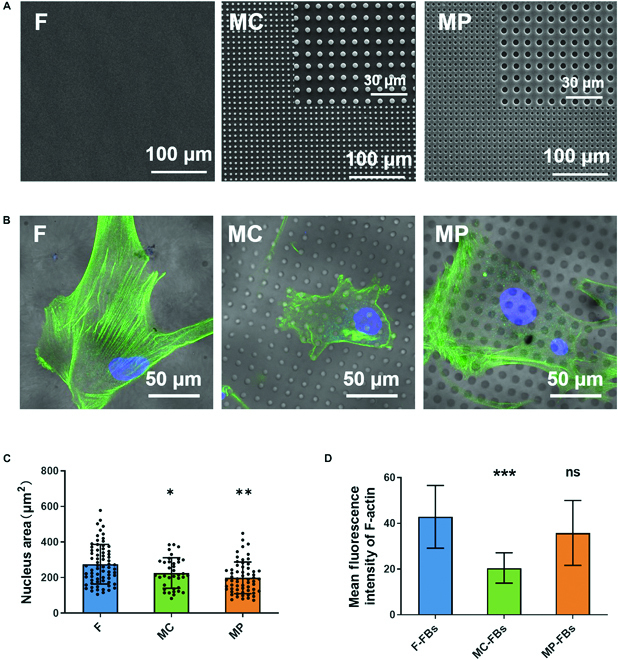
Fibroblasts (FBs) on micropatterned PCL surfaces. (A) Scanning electron microscopy images of PCL surface with no structure (F), micro column (MC), and micro pit (MP). (B) Representative images of FB morphologies. (C) Statistical analysis of the nuclear projection area of FBs. (D) Statistical analysis of the F-actin levels in FBs. *P < 0.05, **P < 0.01, ***P < 0.001. ns, not significant.

From the scanning electron microscopy image of the prepared micropatterned PCL substrates (Fig. 1A and Fig. S1), PCL surfaces embossed with 5-μm-diameter, 6-μm-high MC and 5-μm-diameter, 6-μm-deep MP structures were successfully fabricated in good accordance with the original design. The distance between each MC and MP unit is 5 μm, which is exactly the same in both groups. The flat PCL surface, which was considered the control, is smooth. There are no other impurities on the surfaces of all groups. In addition, human dermal FBs on different micropatterned surfaces showed a similar proliferation rate, indicating that the micropatterns did not affect cell proliferation (Fig. S2).

To observe the morphology of FBs growing on different micropatterned PCL surfaces, the cytoskeleton and nucleus were labeled with F-actin and 4′,6-diamidino-2-phenylindole (DAPI), respectively. The confocal laser images showed that FBs attached and spread normally on all surfaces, although FBs on surfaces with MC had smaller cell areas than those on MP and flat surfaces (Fig. 1B). Further statistical analysis showed that both micropatterns significantly reduced the nuclear projection area of FBs (Fig. 1C). In addition, fewer F-actin bundles were observed in FBs on the surface with micropatterns compared with the control. F-actin fluorescence intensity was also significantly lower in FBs on surfaces with MC, indicating that MC induced less F-actin formation in FBs (Fig. 1D). Similarly, Sunami et al. [34] reported that NIH-3T3 cells on triangular micropatterns with 3- to 20-μm-long sides expressed lower levels of F-actin. A possible explanation for this phenomenon is that surfaces with MC provided limited small anchor points for cells to adhere to and form FAs. As a result, fewer large bundles of F-actin (also known as “stress fiber”), which are anchored at both ends by FAs, can be formed in FBs in the MC group than in the control and MP groups. Compared to the flat and MP surfaces, MC surfaces provide limited anchor points for cells to adhere to.

### PCL surfaces with MC inhibited actin-MRTF signaling in FBs

**Fig. 2.**
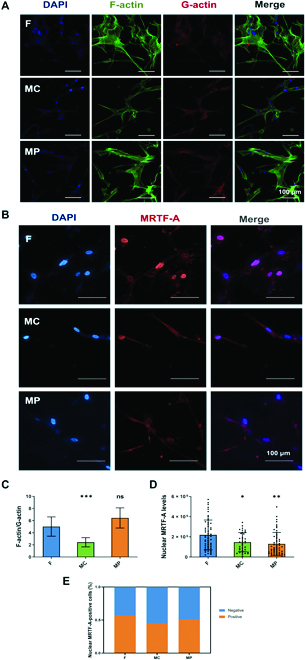
Regulation of actin-MRTF-A signaling in FBs by micropatterned PCL surfaces. (A) Immunofluorescence staining of F-actin and G-actinin FBs. (B) Immunofluorescence staining of MRTF-A in FBs. (C) Ratio of F-actin/G-actin. (D) Statistical analysis of nuclear MRTF-A level in FBs. (E) Statistical analysis of the percentage of MRTF-A-positive cells. *P < 0.05, **P < 0.01, ***P < 0.001. DAPI, 4′,6-diamidino-2-phenylindole.

Since F-actin is assembled by globular actin (G-actin) subunits and the actin pool in cells is maintained at a relatively stable level [35], our results suggest that more G-actin exists in FBs on MC compared to other groups. To validate the regulation of actin dynamics in FBs on micropatterned PCL surfaces, both F-actin and G-actin were visualized through fluorescent staining. As expected, the level of F-actin was markedly decreased in FBs on surfaces with the MC micropattern compared to that of the control and MP groups. Correspondingly, the amount of cytoplasmic G-actin was increased in the MC group (Fig. 2A). Further quantitative measurement demonstrated that the F-actin/G-actin ratio in FBs on MC micropattern surfaces was significantly lower than that in the control and MP groups (Fig. 2C). These results indicated that the MC micropattern triggered the F- to G-actin transition (Fig. 2C).

In response to local stress signals and biomechanical tension, FBs proliferate and eventually differentiate into myofibroblasts [36,37]. MRTFs are serum response factor (SRF) cofactors that have been demonstrated to be key mediators that promote myofibroblastic differentiation [38,39]. MRTF-A is expressed and sequestered in the cytoplasm via interaction with G-actin in FBs. Stimuli (e.g., stress signaling, mechanical force, and change in cell shape) that change actin dynamics and stimulate actin polymerization result in the dissociation of MRTF-A from G-actin and its subsequent nuclear translocation, thereby activating SRF-dependent smooth muscle gene transcription. The elevated level of cytoplasmic G-actin in FBs on surfaces with MC micropatterns suggests the sequestration of MRTF-A. Indeed, significantly reduced nuclear accumulation of MRTF-A was observed in FBs in the MC group (Fig. 2D), which was accompanied by a decrease in the percentage of MRTF-A-positive cells (Fig. 2E). Surprisingly, nuclear MRTF-A was also downregulated in the MP group, in which the level of G-actin was not increased. Our results indicated that surfaces with MC micropatterns inhibited the activation of actin-MRTF-A signaling in FBs.

**Fig. 3.**
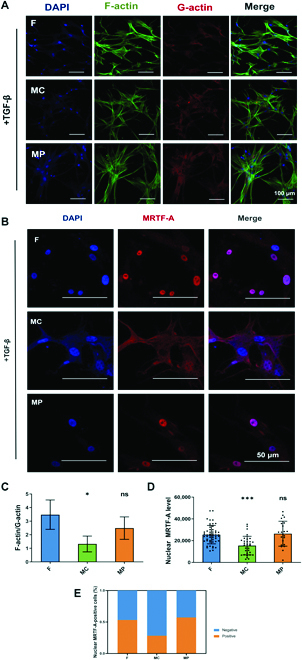
Regulation of actin-MRTF-A signaling in FBs by micropatterned PCL surfaces under transforming growth factor-β (TGF-β) stimulation. (A) Immunofluorescence staining of F-actin and G-actinin FBs. (B) Immunofluorescence staining of MRTF-A in FBs. (C) Ratio of F-actin/G-actin. (D) Statistical analysis of nuclear MRTF-A level in FBs. (E) Statistical analysis of the percentage of MRTF-A-positive cells. *P < 0.05, ***P < 0.001.

Next, the regulation of micropatterned PCL surfaces on the activation of actin-MRTF signaling in FBs in a profibrotic environment was evaluated by treating cells with one of the most potent known fibrogenic factors, transforming growth factor-β (TGF-β). Consistent with the results without TGF-β treatment, MC micropatterned surfaces increased the level of G-actin while decreasing the level of F-actin (Fig. 3A). The MC group also had a considerably lower F-actin/G-actin ratio than the control group (Fig. 3C). Subsequently, the actin-dependent nuclear translocation of MRTF-A and the percentage of MRTF-A-positive cells were inhibited in FBs on MC micropatterned surfaces (Fig. 3D and E). Interestingly, MP micropatterned surfaces did not exhibit similar inhibition of the nuclear MRTF-A level in FBs in the presence of TGF-β. Our results demonstrated that the MC micropatterned surface possessed the ability to inhibit actin-MRTF signaling even in a profibrotic environment.

### PCL surfaces with MC inhibited the myofibroblastic differentiation of FBs

**Fig. 4.**
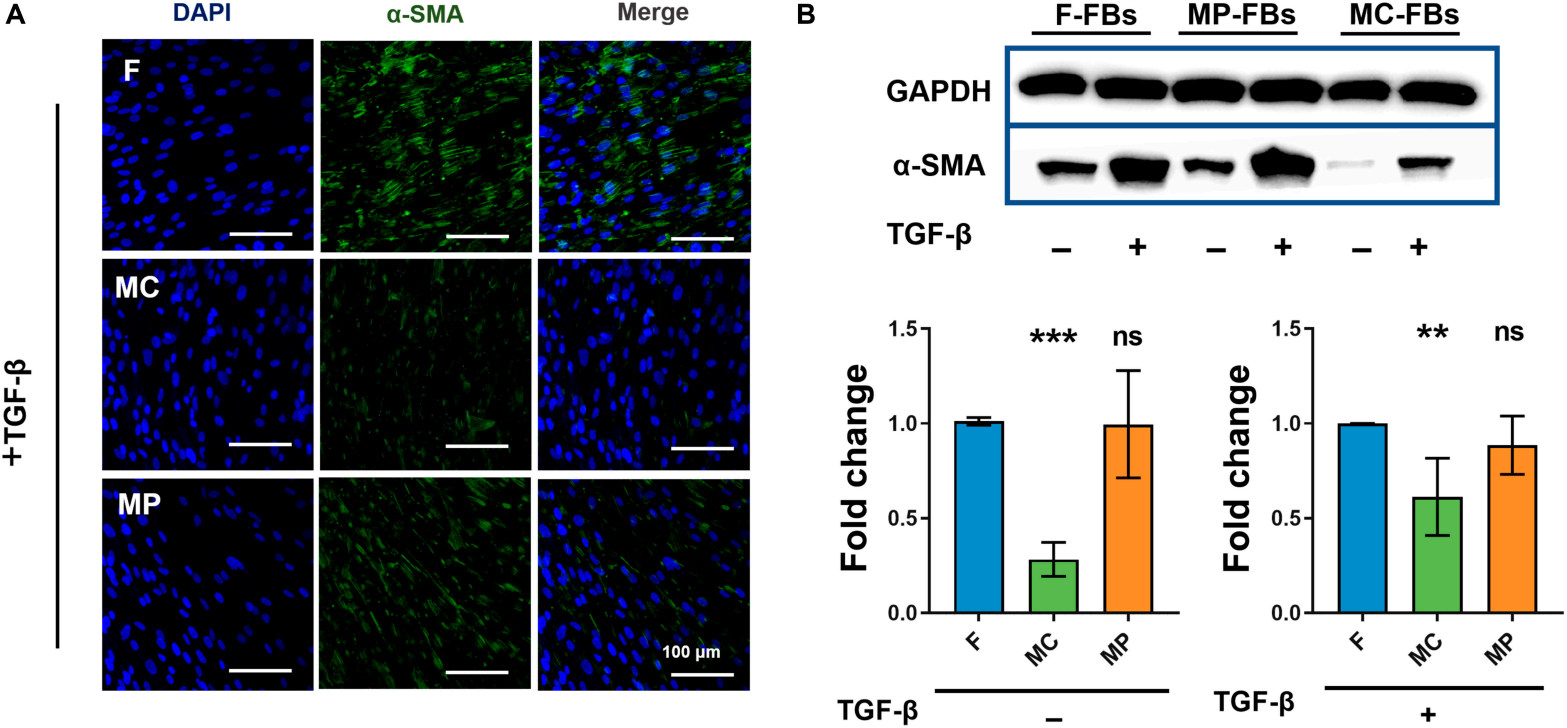
The myofibroblastic differentiation of FBs on micropatterned PCL surfaces. (A) Immunofluorescence staining of α-smooth muscle actin (α-SMA) in FBs. (B) Western blot analysis of α-SMA expression in FBs. **P < 0.01, ***P < 0.001.

The inhibited actin-MRTF signaling in FBs on MC micropatterned surfaces suggests suppression of FB differentiation into myofibroblasts. Thus, the expression of α-SMA, which is the hallmark of mature myofibroblasts, was determined. As shown in Fig. 4A, after stimulation by TGF-β, the majority of FBs on the flat PCL surfaces expressed α-SMA at a high level, while very few FBs on the MC micropatterned surfaces expressed α-SMA. α-SMA expression was also detected in FBs on the MP micropatterned surfaces, albeit at a lower level than that in the control group. The decreased expression of α-SMA in FBs in the MC group was further confirmed by western blotting analysis (Fig. 4B). Apparently, MC micropatterned surfaces inhibited the TGF-β-induced myofibroblastic differentiation of FBs. It is worth mentioning that SRF controls the transcription of many genes, thereby regulating diverse cellular processes, such as cell proliferation, migration, differentiation, and apoptosis [40–42]. Thus, although only FB differentiation was investigated in this study, MC micropatterned surfaces may affect other cell behaviors through actin-MRTF-SRF signaling.

### PCL implants with MC surfaces inhibited fibrosis in vivo

**Fig. 5.**
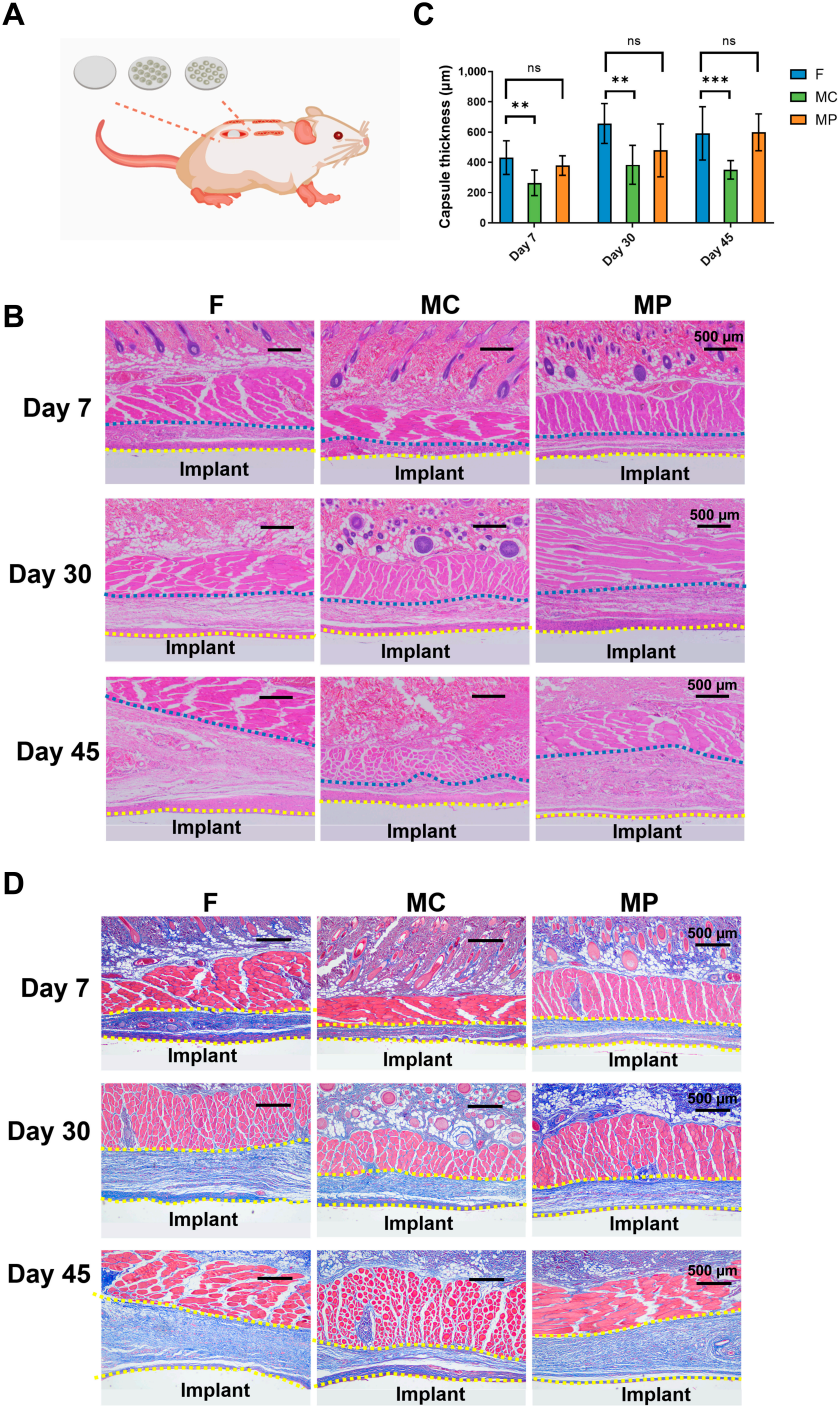
The fibrotic reaction of PCL implants with micropatterned surfaces on days 7, 30, and 45 postsurgery. (A) Schematic diagram of the rat subcutaneous implantation model. (B) H&E staining of peri-implant fibrotic tissue. (C) Statistical analysis of fibrotic layer thickness. (D) Masson staining of peri-implant fibrotic tissue. **P < 0.01, ***P < 0.001.

Compared to simple in vitro culture conditions, implants are exposed to a much more complex environment with numerous stimuli and multiple types of cells that may affect the fibrotic reaction in vivo. Therefore, to test whether PCL surfaces with MCs have antifibrotic effects in vivo, PCL wafers with different surface micropatterns were implanted subcutaneously in rats (Fig. 5A). The peri-implant fibrotic tissue was first visualized via both hematoxylin and eosin (H&E) staining and Masson staining to evaluate the maturation of the fibrotic capsule over time. As shown in Fig. 5, after 7 days of implantation, fibrotic tissue was formed around all implants, but the MC group had thinner fibrotic tissue. With the extension of implantation time, the fibrous capsule gradually thickened (Fig. 5B), with an increasing number of collagen bundles deposited in all groups (Fig. 5D). On the 45th day after implantation, the average thickness of the fibrous capsule formed in the MC group was 350.9 μm, while the other 2 groups had reached more than 590 μm (Fig. 5C). However, it is noted that the thickness of the fibrous capsules from all groups seemed to stop increasing after 30 days postimplantation. During the whole observation period, implants with MC surfaces showed better antifibrotic properties, as evidenced by the lesser thickness of fibrotic capsules compared to implants with flat or MP surfaces (Fig. 5).

**Fig. 6.**
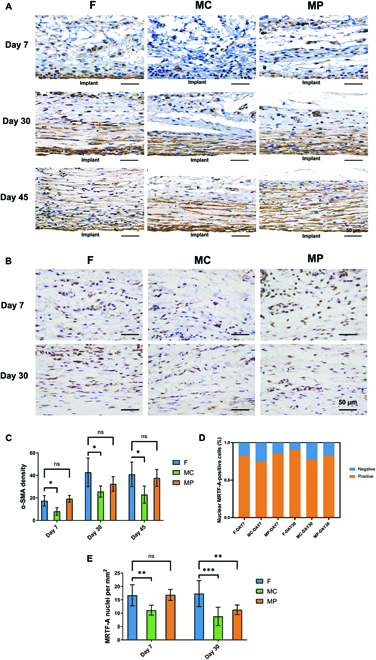
The formation of myofibroblasts in the fibrotic capsule tissue around the implants with micropatterned surfaces. (A) α-SMA immunohistochemical staining on days 7, 30, and 45 postsurgery. (B) MRTF-A immunohistochemical staining on days 7 and 30 postsurgery. (C) Quantitative analysis of α-SMA expression. (D) Quantitative analysis of the percentage of MRTF-A-positive cells. (E) Quantitative analysis of MRTF-A-positive nuclei. *P < 0.05, **P < 0.01, ***P < 0.001.

To detect myofibroblasts in the fibrotic capsule tissue, we performed α-SMA immunohistochemical staining. On day 7, a marked α-SMA-positive signal was detected in the capsule tissue formed around the implants with flat and MP micropatterned surfaces, while a very limited amount of α-SMA was observed in the MC group, indicating that MC surfaces inhibited the transformation of FBs into myofibroblasts (Fig. 6A). Alpha-SMA expression increased gradually with the extension of implantation time. On day 30, α-SMA expression was enhanced in all 3 groups. The α-SMA density in the MC group remained the lowest, and this trend continued for the rest of the observation period (Fig. 6C). No substantial difference in α-SMA density was observed between the MP and control groups. At this moment, α-SMA-expressing myofibroblasts started to exhibit elongated morphologies and arrange in an orderly manner along the surface of the implants. On day 45 after implantation, almost all cells in the control and MP groups in our visual field became α-SMA positive. Thus, the inhibited formation of fibrotic capsules around PCL implants with MC surfaces may be attributed to its inhibitory effects on the myofibroblastic differentiation of FBs.

Tissue cells sense and respond to PCL implants with micropatterned surfaces mainly through mechanotransduction. Thus, to further investigate the underlying mechanism of the inhibited myofibroblastic differentiation by MC surfaces, the nuclear translocation of MRTF-A was determined by immunohistochemical staining (Fig. 6B). On day 7, more MRTF-A-positive cells, together with a larger number of nuclei costained with MRTF-A, were observed in the control and MP groups than in the MC group (Fig. 6D and E), indicating that MC surfaces inhibited the expression of MRTF-A and its nuclear translocation in FBs. On day 30, while the number of MRTF-A-positive cells did not change substantially in all 3 groups, MRTF-A-positive nuclei were notably decreased in both the MC and MP groups compared to the number on day 7 (Fig. 6D and E), which was much lower than that in the control group. These findings suggest that the decreased nuclear translocation of MRTF-A may be involved in limiting the myofibroblastic differentiation of FBs by MC surfaces.

The present study only focused on how micropatterned surfaces affected the myofibroblastic differentiation of FBs. However, other cell types involved in fibrotic reactions may also be capable of sensing micropatterns and responding to them. For example, M2 macrophages secrete large amounts of TGF-β and other profibrotic factors (e.g., connective tissue growth factor, C-C motif chemokine ligand 18, and C-C motif chemokine ligand 17) and are thus considered profibrotic macrophages [43]. Remarkably, numerous studies have shown that macrophages are sensitive to the topographic cues provided by materials. It has been reported that microgrooves induce the M1-to-M2 phenotypic switch of macrophages [44]. Thus, the MC micropattern may affect the process of fibrosis by modulating the phenotype of macrophages as well. Further investigation is required to identify the potential synergistic antifibrotic effects of the MC micropattern.

## Conclusion

**Fig. 7.**
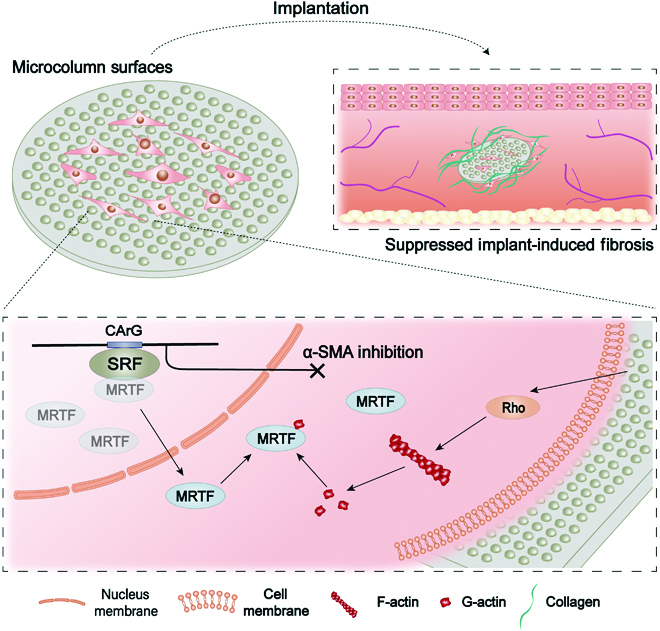
Schematic overview of how micro-column structure affects actin assembly and inhibits MRTF-A nuclear transfer, thereby inhibiting FB differentiation and the formation of implant-induced fibrotic capsules. As indicated in the figure, mechanical stimulation induced the F- to G-actin transition and nuclear transfer of MRTF-A, thereby inhibiting α-SMA expression and FB to myofibroblast differentiation, and in vivo, it caused a reduction in the degree of fibrosis on the implant surface.

In this study, the distinct regulatory effects of typical surface micropatterns, including MC and MP, on the myofibroblastic differentiation of FBs were demonstrated. Compared to the flat and MP surfaces, surfaces with MC induced F- to G-actin transition and suppressed the actin-dependent nuclear translocation of MRTF-A, thereby inhibiting FBs from differentiating into α-SMA-expressing myofibroblasts (Fig. 7). More importantly, further in vivo experiments showed that the PCL implants with MC surfaces also showed an inhibitory effect on the formation of implant-induced fibrotic capsules. The reduced fibrosis by MC surfaces may be attributed to the suppressed myofibroblastic differentiation of FBs.

## Materials and Methods

### Fabrication and characterization of micropatterned PCL surfaces

Micropatterned PCL surfaces were fabricated using photolithography and casting. In simple terms, photolithography was used to prepare silicon wafer molds with MC and MP patterns. Next, a mixture of polydimethylsiloxane (PDMS) base and curing agent (10:1 w/w) (Sylgard, USA) was cast on the master silicon wafer and cured at 90 °C for 1 h to obtain the PDMS master wafer. Then, using the PDMS master as a template, a replica of PCL (molecular weight = 80,000, Sigma-Aldrich, USA) with a designed micropattern was obtained by casting (80 °C, 2 h). Flat PCL surfaces are prepared using a smooth PDMS template. To characterize the micropatterns on the PCL surfaces, field emission scanning electron microscopy (Merlin, Carl Zeiss, Germany) was used.

### Cell culture

Human dermal FBs were cultured in Dulbecco’s modified Eagle’s medium (Gibco, USA) containing 10% fetal bovine serum (Gibco) at 37 °C under 5% CO_2_. To investigate the effects of micropatterned surfaces on FBs, cells were seeded on the surface of micropatterned PCL at a density of 3 × 10^4^ cells/cm^2^ and cultured for more than 24 h. TGF-β (10 ng/ml) was added to the medium to induce the differentiation of FBs.

### Immunofluorescence staining

Samples were fixed with 4% paraformaldehyde and then permeabilized with 0.5% Triton. After being blocked with 5% bovine serum albumin, the samples were incubated with the following reagents: YF®488-phalloidin (US EVERBRIGHT, China, 1:200), anti-α-SMA (Abcam, UK, 1:500), and anti-MRTF-A (Thermo Fisher, USA, 1:200). For the primary antibodies without conjugated fluorescent labels, samples were further incubated with goat anti-rabbit IgG H&L Alexa Fluor® 647 secondary antibody (Abcam, 1:1,000). Cell nuclei were stained with DAPI (Beyotime, China). The staining was observed with a confocal laser scanning microscope (LSM880 WITH AIRYSCAN).

### Western blotting

FBs (9.6 × 10^4^) were seeded on micropatterned PCL trimmed to the same size as the microwell of 6-well plates. Cells were lysed in radio immunoprecipitation assay buffer (Beyotime) on ice. After centrifugation at 12,000 × g, protein lysates were obtained. After determining the protein concentration using a bicinchoninic acid kit (Beyotime), equal amounts of protein samples were separated by 10% sodium dodecyl sulfate-polyacrylamide gel electrophoresis (Bio-Rad, USA) and then electrotransferred to polyvinylidene fluoride membranes. The membranes were blocked with 5% skimmed milk and incubated with either anti-glyceraldehyde phosphate dehydrogenase (Signalway, 1:5,000) or anti-α-SMA (Abcam, 1:1,000) overnight at 4 °C. Next, the membrane was washed and incubated with horseradish peroxidase-conjugated secondary antibody (Signalway, 1:5,000) with shaking at room temperature for 1 h. High-sig enhanced chemiluminescence western blotting substrate (Tanon, China) was used for the color development reaction. Imaging was observed on a Chemidoc^TM^ MP Imaging System (Bio-Rad). The grayscale values of the bands were analyzed using ImageJ. Glyceraldehyde phosphate dehydrogenase was used as the internal reference.

### Subcutaneous implantation of PCL wafers

All animal procedures were approved by the Animal Care and Use Committee of South China University of Technology. Twelve specific pathogen-free Sprague-Dawley rats with a body weight of 200 to 220 g were obtained from the Laboratory Animal Research Center of South China University of Technology and kept in the specific pathogen-free barrier environment throughout the whole period. After the rats were anesthetized by inhaling isoflurane, four 1-cm incisions were created with a scalpel on the dorsal back. The subcutaneous skin of the back is separated from the muscle using blunt-ended forceps to create a pocket. Double-sided PCL wafers with different micropatterned surfaces were randomly placed into the subcutaneous pocket. Each subcutaneous pocket held only 1 PCL wafer, and they were completely separated from each other. After implantation, the incisions were sutured. The rats were sacrificed with a CO_2_ asphyxiation machine at 7, 30, and 45 d postsurgery, and the implanted PCL wafers together with the fibrotic capsules were harvested for further analysis.

### Histological analysis and immunohistochemical staining

The harvested samples were first fixed with 4% paraformaldehyde. The fixed samples were then dehydrated in a graded ethanol series, embedded in paraffin wax, and sliced into 1-mm-thick sections. Afterward, the sections were dewaxed in xylene and rehydrated in a graded ethanol series. Finally, the sections were stained with H&E and Masson staining.

For immunohistochemical staining, the sectioned samples were first dewaxed and rehydrated. Then, they were incubated in EDTA antigen repair buffer for antigen repair. Immunohistochemical staining was performed as previously described [45]. In simple terms, the samples were immersed in 3% hydrogen peroxide solution, blocked with 5% bovine serum albumin, and incubated with anti-α-SMA (Abcam, 1:1,000) and anti-MRTF-A (Thermo Fisher, 1:2,000) overnight. After washing, the samples were incubated with horseradish peroxidase-conjugated secondary antibody. The antibody binding sites were visualized by incubation with diaminobenzidine. Harris hematoxylin was used for nuclear staining.

### Statistical analysis

Three independent experiments were performed with at least triplicates per group. GraphPad Prism 8 software (GraphPad) was used for statistical analysis. All data are expressed as the mean ± standard deviation (SD). Statistical significance was determined using a *t* test. A value of *P* < 0.05 was considered to be statistically significant.

## Data Availability

The data that support the findings of this study are available from the corresponding author upon reasonable request.
